# Phase I study of oral ridaforolimus in combination with paclitaxel and carboplatin in patients with solid tumor cancers

**DOI:** 10.1186/s12885-017-3394-2

**Published:** 2017-06-08

**Authors:** Hye Sook Chon, Sokbom Kang, Jae K. Lee, Sachin M. Apte, Mian M. Shahzad, Irene Williams-Elson, Robert M. Wenham

**Affiliations:** 1 Department of Gynecologic Oncology, H. Lee Moffitt Cancer Center and Research Institute, 12902 Magnolia Drive, Tampa, FL 33647 USA; 2 Division of Gynecologic Cancer Research, Center for Uterine Cancer, National Cancer Center, Ilsan-gu Madu-dong, Goyang, 410-768 Korea; 3 Department of Biostatistics and Bioinformatics, H. Lee Moffitt Cancer Center and Research Institute, 12902 Magnolia Drive, Tampa, FL 33647 USA; 4 Clinical Trials Office, Phase 1 Clinical trials, H. Lee Moffitt Cancer Center and Research Institute, 12902 Magnolia Drive, Tampa, FL 33647 USA

**Keywords:** Oral ridaforolimus, Phase 1 trial, Paclitaxel and carboplatin combination, Solid tumors

## Abstract

**Background:**

Ridaforolimus is a mammalian target of rapamycin inhibitor that has activity in solid tumors. Paclitaxel and carboplatin have broad antineoplastic activity in many cancers. This phase I trial was conducted to determine the safety profile, maximal tolerated dose, and recommended phase II dose and schedule of oral ridaforolimus combined with paclitaxel and carboplatin in patients with solid tumor cancers.

**Methods:**

Eligible patients with advanced solid tumor cancers received oral 10 to 30 mg ridaforolimus daily for 5 consecutive days per week combined with intravenous paclitaxel (175 mg/m^2^) and carboplatin (area under the curve [AUC] 5–6 mg/mL/min) in 3-week cycles. A standard 3 + 3 design was used to escalate doses, with predefined changes to an alternate dosing schedule and/or changes in carboplatin AUC doses based on dose-limiting toxicity (DLT). Secondary information was collected regarding response and time to progression. Patients were continued on treatment if therapy was tolerated and if stable disease or better was demonstrated.

**Results:**

Thirty-one patients were consented, 28 patients were screened, and 24 patients met eligibility requirements and received treatment. Two patients were replaced for events unrelated to drug-related toxicity, resulting in 22 DLT-evaluable patients. Two grade 4 DLTs due to neutropenia were observed at dose level 1. The next cohort was changed to a predefined alternate dosing schedule (days 1–5 and 8–12). DLTs were neutropenia, sepsis, mucositis, and thrombocytopenia. The most common adverse events were neutropenia, anemia, thrombocytopenia, fatigue, alopecia, nausea, pain, and leukopenia. Twenty-four patients received a median of 4 cycles (range, 1–12). Evaluable patients for response (*n* = 18) demonstrated a median tumor measurement decrease of 25%. The best response in these 18 patients included 9 patients with partial response (50%), 6 with stable disease (33%), and 3 with progressive disease (17%). Thirteen of these patients received treatment for 4 or more cycles.

**Conclusions:**

Treatment with ridaforolimus combined with paclitaxel and carboplatin had no unanticipated toxicities and showed antineoplastic activity. The recommended phase II dose and schedule is ridaforolimus 30 mg (days 1–5 and 8–12) plus day 1 paclitaxel (175 mg/m^2^) and carboplatin (AUC 5 mg/mL/min) on a 21-day cycle.

**Trial registration:**

ClinicalTrials.gov Identifier: NCT01256268 (trial registration date: December 1, 2010).

## Background

Because mammalian target of rapamycin (mTOR) inhibitors target the downstream effects of the PI3K/AKT/PTEN-related pathways, this class of drugs has broad antiproliferative activity [1]. Ridaforolimus (deforolimus; AP23573; MK 8669, AP 23573), a potent mTOR inhibitor with an IC_50_ in the nanomolar range, appears to be well tolerated in both intravenous and oral formulations as either a single agent or in combination with other chemotherapy agents [2]. In preclinical studies, ridaforolimus demonstrated antitumor activity against a broad range of human cancer cell lines in vitro and tumor xenograft models in vivo [3–6]. In phase I and II clinical trials, ridaforolimus displayed activity in various cancers, including sarcoma and hematologic malignancies [7–10]. In a phase III trial of patients with advanced sarcoma, single-agent ridaforolimus treatment (40 mg orally, once daily for 5 consecutive days every week) resulted in a statistically significant improvement in progression-free survival compared with placebo [11]. Ridaforolimus has shown additive or synergistic activity when combined with other single agents, such as paclitaxel, carboplatin, cisplatin, doxorubicin, imatinib, and trastuzumab [12, 13]. Therefore, combining chemotherapy regimens with an mTOR inhibitor with a different mechanism of action and reasonable toxicity may provide an advantageous clinical approach.

The combination of paclitaxel and carboplatin is one of the most commonly used chemotherapeutic combinations in cancer treatment, including head and neck cancer, advanced-stage non-small cell lung cancer, endometrial cancer, ovarian cancer, and others. Oral ridaforolimus has shown equivalent effectiveness comparable to the intravenous form [14]. Therefore, the potential benefit of a convenient oral dosing with paclitaxel plus carboplatin warranted investigation. In this phase I study, our aim was to determine the maximal tolerated dose (MTD) and the recommended phase 2 dose and schedule of oral ridaforolimus in combination with paclitaxel and carboplatin in patients with solid tumor cancers and to describe the safety and tolerability of this combination.

## Methods

### Study eligibility

Patients ≥18 years of age with solid tumor cancers not deemed curable by other therapies and who had measurable disease by Response Evaluation Criteria in Solid Tumor (RECIST) 1.1 or evaluable disease were eligible. Other eligibility criteria included Eastern Cooperative Oncology Group performance status of 0, 1, or 2; a life expectancy of at least 60 days; adequate bone marrow function, renal function, hepatic function, and neurologic function; serum cholesterol ≤350 mg/dL and triglyceride ≤400 mg/dL; and full recovery to baseline from acute toxicities of all prior chemotherapy regimens. Patients may have had up to 3 (0–3) prior cytotoxic chemotherapeutic regimens including prior treatment with carboplatin and paclitaxel (patients who had regimens switched for toxicity rather than progression, used for radiation sensitization only, or hormonal only were not eligible). No chemotherapy, radiotherapy, biologic, hormonal, or investigational drug therapy within 28 days before start of study treatment was permitted. Patients were excluded if they had any upper gastrointestinal illness that would impair swallowing or absorption of oral medication, any intercurrent illness, were known to have human immunodeficiency virus or AIDS, had received prior therapy with an mTOR inhibitor, or had concomitant treatment with inhibitors or inducers of cytochrome P450-3A. The study protocol was approved by the University of South Florida Institutional Review Board. All patients provided written informed consent before study participation.

### Study design and treatment

Patients received oral ridaforolimus daily on days 2–5, days 8–12, and days 15–19 during the first cycle of therapy and then 5 days a week (days 1–5, days 8–12, and days 15–19) throughout the remainder of therapy beginning with the second cycle of therapy. Oral ridaforolimus was administered in combination with day 1 intravenous paclitaxel (175 mg/m^2^) and carboplatin (AUC = 5–6 mg/mL/min) every 3 weeks, except for the first cycle of therapy where day 1 ridaforolimus was skipped to allow for blood samples to be collected day 1 of the first 2 cycles. These were held for potential PK analyses if specific drug and temporally related toxicities were noted. All patients received steroids, antiemetics, and antihistamines before the administration of paclitaxel and carboplatin. All patients were expected to continue study treatment in the absence of disease progression, complete response, unacceptable toxicity, or voluntary choice to withdraw participation. A 3 + 3 dose escalation design was used, with ridaforolimus dose levels of 10, 20, 30, and 40 mg orally in combination with intravenous paclitaxel and carboplatin based on a predefined dose escalation scheme. Carboplatin was dosed at an AUC of 5, with a planned escalation to an AUC of 6 mg/mL/min based on dose level cohort.

The maximal tolerated dose was defined as the highest dose at which no more than 1 of 6 evaluable patients experienced a dose-limiting toxicity (DLT) due to the combination of ridaforolimus, paclitaxel, and carboplatin during the first cycle of treatment. A patient who did not complete the first cycle of treatment for reasons other than a DLT was replaced. DLT was defined as ≥ grade 3 non-hematologic toxicity (specifically, rash, mucositis, pneumonitis) with the exceptions of fatigue, hypersensitivity reaction, nausea, and vomiting; ≥ grade 3 thrombocytopenia requiring platelet transfusion; grade 4 thrombocytopenia or neutropenia >7 days duration; any grade 4 neutropenic fever requiring hospitalization; unresolved toxicity resulting in delay of retreatment >2 weeks; grade 3 or 4 non-surgical hemorrhages; and failure of administration of ridaforolimus for 5 days or more (consecutive or nonconsecutive) due to any toxicity. Growth factor support was not allowed prophylactically for cycle 1 but could be subsequently used based on investigator discretion.

A modification of the schedule that changed ridaforolimus administration to the first 2 weeks (days 1–5, days 8–12) versus all 3 weeks (days 1–5, days 8–12, days 15–18) of a cycle was predefined for 2 DLTs in a cohort that resulted from thrombocytopenia or neutropenia in the latter part of the cycle. The dose of ridaforolimus remained the same as the maximum achieved level in the prior cohort at which the DLTs were experienced. Dose escalation was to continue at each subsequent cohort until a maximum of 40 mg/day (days 1–5, days 8–12) of ridaforolimus was reached. Subsequent treatment cycles would not begin until absolute neutrophil count reached ≥1500 cells/mm^3^ and platelet count reached ≥75,000/mm^3^; mucositis, nausea, and vomiting were grade 1 or less; and bilirubin was ≤1.5 x institutional upper limit of normal. All drugs were held during the recovery period. Therapy was delayed for a maximum of 2 weeks until these values were achieved. Patients who failed to recover adequate counts within a 2-week delay were removed from study. Adverse events were graded according to the Common Terminology Criteria for Adverse Events version 4.0.

### Efficacy and safety assessments

Patients were evaluated at baseline and before each subsequent treatment cycle to assess Eastern Cooperative Oncology Group performance status, vital signs, and adverse events. Hematologic and clinical chemistry assessments, including cholesterol, triglyceride, and glucose levels, were performed at baseline and at each treatment cycle. Tumor assessment by RECIST v1.1 was performed at baseline and every 2 cycles thereafter. Patients were required to have completed a minimum of 2 cycles of therapy to be evaluable for efficacy.

## Results

### Patients and study treatment

Thirty-one patients were consented and 24 patients were enrolled between June 2011 and May 2014. A total of 116 cycles were initiated until January 2015. All patients who received at least one dose of study medication were included in the toxicity analyses (*n* = 24 patients). Two patients were replaced for DLT evaluation (see below). Patients who completed required imaging after the second cycle were included for efficacy analyses (*n* = 18 patients). The mean age was 62 years (range, 30–72 years), and the median number of prior chemotherapy treatments was 2 (range, 0–3). Tumor types included ovarian/fallopian/primary peritoneal (*n* = 10), endometrial (*n* = 5), cervical (*n* = 3), esophageal (*n* = 2), and urethral, vaginal, mesothelial, and salivary (*n* = 1 for each). Number of cycles delivered to patients ranged from 1 to 12 (median of 5 cycles; *n* = 22 patients evaluable for DLT). Patient characteristics are summarized in Table 1.

**Table 1:** Patient Characteristics (*n* = 24)

Characteristics	No. of Patients (%)
Age, years	
Median	62
Range	30–72
Sex	
Male	4 (17)
Female	20 (83)
Race	
White	22 (90)
Black	0
Other	2 (10)
ECOG performance status	
ECOG 0	16 (67)
ECOG 1	7 (29)
ECOG 2	1 (4)
Tumor type	
Ovarian/fallopian/peritoneal	10 (42)
Endometrial	5 (21)
Cervical	3 (13)
Esophageal	2 (8)
Urethral	1 (4)
Vaginal	1 (4)
Mesothelial	1 (4)
Salivary	1 (4)
Prior chemotherapies^a^	
0	7 (29)
1	1 (4)
2	7 (29)
3	9 (38)

*ECOG* Eastern Cooperative Oncology Group

^a^Therapies that included chemotherapy for radiation sensitization only (*n* = 4), were discontinued due to toxicity without progression (*n* = 2), were radiation alone (*n* = 4), or were hormonal only (*n* = 2) were not included for eligibility

### Safety

The number of patients enrolled and evaluable at each dose level and the DLTs are summarized in Table 2. Two DLTs of grade 4 neutropenia were observed at dose level 1 (ridaforolimus 10 mg from days 1–5, days 8–12, and days 15–18 combined with 175 mg/m^2^ paclitaxel and carboplatin (AUC 5 mg/mL/min)). A predefined alternate dosing cohort (days 1–5, days 8–12) was opened at the same dose of ridaforolimus (dose level 1A). There was one DLT with sepsis at alternate dose level 1A (1 of 6 patients). No DLTs were observed at alternate dose levels 2A (20 mg ridaforolimus) and 3A (30 mg ridaforolimus). Dose escalations were continued to cohort 4A (30 mg ridaforolimus, 175 mg/m^2^ paclitaxel, and AUC = 6 mg/mL/min carboplatin). At dose level 4A, 2 of 6 patients had DLTs (1 grade 3 mucositis and 1 grade 4 thrombocytopenia). Thus, the maximal tolerated dose was established as the 3A dose level (30 mg ridaforolimus combined with 175 mg/m^2^ paclitaxel and AUC = 5 mg/mL/min carboplatin). Two patients were replaced for DLT determination during cycle 1 (1 patient from cohort 2A for noncompliance and 1 patient from cohort 4A due to *C. difficile* diarrhea and diverticulitis deemed unrelated to treatment). Allowed drug-specific dose reductions after cycle 1 were done in 9 patients after a median of 5 cycles, including for paclitaxel + carboplatin (*n* = 4), paclitaxel only (*n* = 3), and ridaforolimus (*n* = 2).

**Table 2:** Patients treated and DLTs by dose level

Dose Level	Ridaforolimus mg (days of cycle)	Carboplatin (AUC)	No. of Patients Enrolled	No of Patients evaluable for DLT^a^	Dose-Limiting Toxicity
1	10 (days 1–5, 8–12, 15–19)	5	4	4	Two grade 4 neutropenia
1A	10 (days 1–5, 8–12)	5	6	6	Death from sepsis
2A	20 (days 1–5, 8–12)	5	4^a^	3	None
3A	30 (days 1–5, 8–12)	5	3	3	None
4A	30 (days 1–5, 8–12)	6	7^b^	6	Grade 3 mucositis; grade 4 thrombocytopenia requiring transfusion

Slots replaced due to ^a^protocol non-compliance or ^b^non-treatment related issue

Paclitaxel was at 175 mg/m^2^ for all cohorts

Treatment-related adverse events observed in >20% of patients are shown in Table 3. The most common adverse events were neutropenia, anemia, thrombocytopenia, fatigue, alopecia, nausea, pain, and leukopenia. The most common grade 3 and grade 4 adverse events were hematologic, including neutropenia (92% of patients: 10 with grade 3 and 12 with grade 4), anemia (42%: 10 with grade 3), thrombocytopenia (67%: 8 with grade 3 and 8 with grade 4), and leukopenia (42%: 8 with grade 3 and 2 with grade 4). Non-hematologic grade 3 and 4 adverse events were infrequent, except hypokalemia (13%; 3 with grade 3 and 1 with grade 4). Of note, two grade 3 hyperglycemia and one grade 3 mucositis were among the less frequent (<10%) high-grade non-hematologic toxicities. One patient with recurrent fallopian tube cancer died. She initiated treatment 7 days before she presented with fever, chills, and abdominal pain. An autopsy showed the cause of death due to multiorgan failure, attributed to sepsis. With no other obvious cause, this was deemed at least possibly related to treatment. Table 4 demonstrates drug-related toxicities by dose level.

**Table 3:** Number of cycles and patients with treatment-related adverse events in >20% of patients (*N* = 24 patients)

	Grade (number of cycles)				Patients	
	1	2	3	4	Number	%
Alkaline phosphatase increased	6				5	21%
Dysphagia	2	2	1		5	21%
Dyspnea	5	3	2		5	21%
Hypoalbuminemia	3	1	1		5	21%
Dehydration	3	5	1		7	29%
Fever	8				7	29%
Hypokalemia	11	1	4	2	7	29%
Transaminases increased	11				7	29%
Hypertriglyceridemia	7	1	1		8	33%
Peripheral sensory neuropathy	8	3			8	33%
Vomiting	7	1	4		8	33%
Anorexia	7	4			9	38%
Urinary tract infection	1	6	2		9	38%
Diarrhea	8	6	1		10	42%
Hyperglycemia	18	6	2		10	42%
Hypomagnesemia	23	6			10	42%
Mucositis oral	10	10	2		11	46%
White blood cell decreased	14	14	29	3	12	50%
Nausea	17	4	3		13	54%
Pain	7				13	54%
Alopecia	8	12			14	58%
Fatigue	14	14	1		15	63%
Anemia	21	45	15		20	83%
Platelet count decreased	32	26	14	14	20	83%
Neutrophil count decreased	8	19	26	20	21	88%

Toxicities by grade seen in >20% of patients deemed possibly, probably, or definitely related in all patients eligible for toxicity evaluation. Under grade, this is listed as: Total Number of Cycles. Under Patients, this is listed as the: Total Number of Patients for any grade. There were 24 patients who received at least 1 dose of treatment and were part of the toxicity evaluation. A patient may be counted only once for each grade of toxicity but may appear under more than one grade for each toxicity

**Table 4:** Drug-related toxicities by dose level for *N* = 24 Patients

	*Level 1*: RIDA 10 mg; P 175 mg/m2; C 5 (*n* = 4)					*Level 1A*: RIDA 10 mg; P 175 mg/m2; C 5 (*n* = 6)					*Level 2A*: RIDA 20 mg; P 175 mg/m2; C 5 (*n* = 4)					*Level 3A*: RIDA 30 mg; P 175 mg/m2; C 5 (*n* = 3)					*Level 4A*: RIDA 30 mg; P 175 mg/m2; C 6 (*n* = 7)				
	Grade				Total No. of Patients	Grade				Total No. of Patients	Grade				Total No. of Patients	Grade				Total No. of Patients	Grade				Total No. of Patients
	1	2	3	4		1	2	3	4		1	2	3	4		1	2	3	4		1	2	3	4	
Alkaline phosphatase increased	2				2	1				1						1				1	1				1
Alopecia		1			1	1	3			4		3			3	2	2			3	2	2			2
Anemia	2	2	3		4	3	4			4	3	4	2		4	1	2			2	3	4	5		6
Anorexia						1	1			2	1				1		2			2	4				4
Dehydration											1				1	1	3			3	1	2	1		3
Diarrhea		1			1	1	1			2	2	1			3						3	1	1		4
Dysphagia						1				1		1			1						1	1	1		3
Dyspnea	1				1			2		2											2	1			2
Fatigue	2	2			2	1	2			3	1	2			2	2	2			3	3	3	1		5
Fever	2				2						1				1	1				1	3				3
Hyperglycemia		1	1		1	2	2			2	2		1		2		1			1	2	2			3
Hypertriglyceridemia		1	1		1	2				2	2				2						3				3
Hypoalbuminemia	1	1			2	2		1		3															
Hypokalemia						1				1	2	1	1	1	2	1				1	2		2		3
Hypomagnesemia		1			1	2				2	2	1			2	1				1	4	2			4
Mucositis oral	1	1			2						2	2			3	2	1			2	3	3	1		4
Nausea	1				1	1				1	1	2	1		3	2	2			3	5		1		5
Neutrophil count decreased	1	1	2	2	4	4	2		4	5		1	3	1	3		2	1	2	3	2	4	4	3	6
Pain	1				1						2				2						3				3
Peripheral sensory neuropathy	1	1			1	3	1			3		1			1	1				1	2				2
Platelet count decreased	1	4			4	3		1	1	3	4	3	2	1	4	1	1	1	2	3	3	4	4	4	6
Transaminases increased											1				1	3				3	1				1
Urinary tract infection		1	1		2												1			1	1	3	1		4
Vomiting	1				1	2				2			1		1	1				1	1	1	1		3
White blood cell decreased						4	2	4		5	1	2	2	1	3				1	1	1	2	2		3

*RIDA* Ridaforolimus, *P* paclitaxel, *C* carboplatin

### Efficacy

**Fig. 1:**
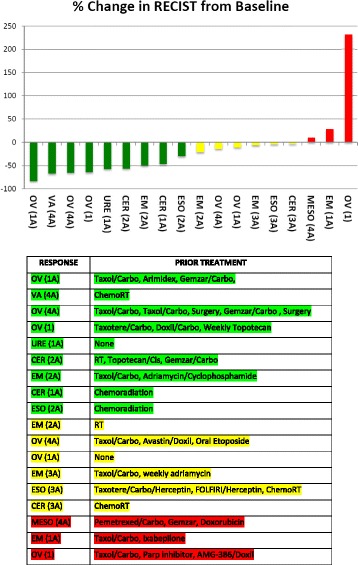
Best response by Response Evaluation Criteria in Solid Tumor (RECIST), measured as maximum percent change of tumor RECIST measurements from baseline. Tumor type (and cohort) are denoted below each bar. Green denotes partial response, yellow denotes stable disease, and red denotes progressive disease. A, alternate dose schedule; CER, cervical; EM, endometrial; ESO, esophageal; MESO, mesothelial; OV, ovarian/fallopian/peritoneal; URE, urethral; VA, vaginal

Eighteen of 24 patients were evaluable for antitumor response (5 were not evaluable because of DLTs and 1 patient was replaced due to discontinuation unrelated to treatment). Best response included 9 patients with partial response (50%), 6 with stable disease (33%), and 3 with progressive disease (17%). In 18 patients, 15 (83%) had stable disease or partial response at the time of first tumor assessment. Thirteen patients received 4 or more treatment cycles (range, 1–12). In the 18 patients evaluable for best response, 6 patients came off study before progression of disease was determined by RECIST. Of the remaining12 patients with RECIST-determined progressive disease, the median duration of response was 81 days (range, 0–236 days) and median time to progression from start of therapy was 166 days (range, 42–393 days). Five patients with partial response or stable disease discontinued treatment due to patient choice; however, these patients were deemed to have no treatment-defined toxicities at the time of discontinuation. One patient remained on treatment for 4 cycles with a partial response but was replaced at cycle 1 as a DLT determination due to noncompliance with drug schedule. The 18 evaluable patients demonstrated a median RECIST 1.1 tumor size decrease of 25% as the best response in target lesion (range, −83% to 232%; Fig. 1). Notably, responses among the 3 cervical and 1 vaginal cancer patients included 1 stable disease and 3 partial responses with a total of 29 cycles (median of 8) delivered. Figure 1 demonstrates best response by RECIST. As shown in Fig. 1, the majority of patients with partial response or stable disease had received prior paclitaxel and carboplatin or carboplatin-based chemotherapy.

## Discussion

This phase I study of ridaforolimus combined with paclitaxel and carboplatin demonstrated tolerability at the defined maximal tolerated dose using doses of the 3 agents considered active in patients with solid tumor cancers. Treatment with ridaforolimus showed toxicities that were expected from its known profile. Mouth sores, rash, fatigue, stomatitis, and hypertriglyceridemia have been most prevalent in phase I and II clinical trials with ridaforolimus as a single agent, with incident rates ranging from 31% to 48% [7, 9]. Previous phase I and II studies have explored combinations of ridaforolimus with capecitabine [15], weekly paclitaxel [16], bevacizumab [16, 17], dalotuzumab [18, 19], and traztuzumab [20] and have demonstrated tolerability. Doses of up to 40 mg ridaforolimus once daily as a single agent for 5 consecutive days with 2 days rest each week have been shown to be tolerable in patients with metastatic or advanced solid tumors [14, 20]. When weekly intravenous ridaforolimus was combined with weekly paclitaxel, 2 recommended doses were determined: 37.5 mg ridaforolimus +60 mg/m^2^ paclitaxel and 12.5 mg ridaforolimus +80 mg/m^2^ paclitaxel [16]. At these recommended doses, a DLT of mucositis was observed, with grade 3/4 neutropenia shown in 14% to 37.5% of the cohorts. In our study, hematologic adverse events were somewhat more prominent, likely because of the nature of combination with two cytotoxic chemotherapies. Non-hematologic adverse events shown in our study were similar to other trials with single-agent ridaforolimus. We had anticipated that the use of these three agents together would have greater potential for bone marrow suppression, namely neutropenia and thrombocytopenia. Therefore, we had preplanned an alternate dosing schedule that shortened the administration of ridaforolimus to 2 weeks (10 days) instead of 3 weeks (15 days). Indeed, the two DLTs of grade 4 neutropenia were observed at the starting dose level of 10 mg ridaforolimus (days 1–5, days 8–12, and days 15–18) combined with paclitaxel (175 mg/m^2^) and carboplatin (AUC = 5 mg/mL/min). An alternate dosing cohort (dose level 1A; days 1–5 and days 8–12) was initiated at the same dose of ridaforolimus as the first cohort. This alternate dosing cohort (2 weeks on and 1 week off) was feasible for repeated cycles. This is similar to the results of the weekly paclitaxel study above in that patients had to switch from intravenous ridaforolimus in the latter part of the cycle (days 8 and 15) to earlier in the cycle (days 1 and 8) [16]. It appears that this earlier cycle dosing is sometimes necessary to allow sufficient marrow recovery when combined with cytotoxic chemotherapy.

Ridaforolimus has activity in cancer, particularly in disease stabilization in various tumor types. In a phase III trial of 702 patients with advanced metastatic sarcoma who had attained benefit with prior chemotherapy, administration of oral ridaforolimus as maintenance therapy resulted in a statistically significant improvement of 3.1 weeks in progression-free survival compared with placebo (hazard ratio of 0.72; 95% confidence interval, 0.61–0.85; *P* = 0.001) [11]. Various mTOR inhibitors, including everolimus (RA001), temsirolimus (CCI779), and ridaforolimus (AP2357), either as a single agent or combined with other chemotherapeutic or hormonal agents have been evaluated in patients with advanced or recurrent endometrial cancer with promising results [21–26]. Mutations or loss of function in PTEN (phosphatase and tensin homolog) plays a significant role in the pathogenesis of endometrial cancer. Downstream activation of the PI3K/AKT/mTOR signaling pathway triggered by the loss of function of PTEN suggests a therapeutic role of the mTOR inhibition. Paclitaxel plus carboplatin is a widely used regimen for this cancer; therefore, it would be of interest to study this combination with ridaforolimus at our recommended phase II dose and schedule in this disease. We also noted interesting activity in patients with cervical and vaginal cancer in our study. Among 3 patients with cervical cancer (1 with adenocarcinoma, 1 with squamous cell carcinoma) and 1 patient with vaginal cancer (squamous cell carcinoma), there were a total 29 cycles of treatment with 1 stable disease and 3 partial responses.

In a preclinical study, Molinolo et al. demonstrated that mTOR pathway activation was shown in most human papillomavirus-positive head and neck squamous cell carcinoma and cervical cancer squamous cell carcinoma tumor xenografts. mTOR inhibitors (rapamycin and everolimus) effectively decreased mTOR activity in vivo and caused a remarkable decrease in tumor burden (*P* < 0.001) [27]. In a phase II study of temsirolimus in patients with recurrent or metastatic cervical cancer, among 33 evaluable patients, 1 patient (3.0%) had a partial response and 19 patients (57.6%) had stable disease with a duration of 6.5 months [28]. There are trials in head and neck squamous cell carcinoma with rapamycin therapy and adding everolimus to definitive chemoradiation treatment in patients with locally advanced cervical cancer. Paclitaxel combined with carboplatin is also a regimen used for the treatment of these cancers; therefore, the addition of ridaforolimus to this combination may be considered for further study, perhaps with or without bevacizumab [29]. In our study, the combination of oral ridaforolimus with intravenous paclitaxel and carboplatin had no unanticipated toxicities with antitumor activity in patients with solid tumor cancers. Given the broad activity and use of paclitaxel and carboplatin in many tumor types, there is potential to explore this triplet therapy in multiple tumors in which mTOR inhibition may be relevant.

## Conclusions

Treatment with ridaforolimus in combination with paclitaxel and carboplatin had no unanticipated toxicities and showed antineoplastic activity. The recommended phase II dose and schedule is ridaforolimus 30 mg (days 1–5 and 8–12) plus day 1 paclitaxel (175 mg/m^2^) and carboplatin (AUC 5 mg/mL/min) on a 21-day cycle. There is potential to explore triplet therapy with ridaforolimus combined with paclitaxel and carboplatin in multiple tumors where mTOR inhibition is relevant.

## Abbreviations

AUC: Area under the curve
DLT: Dose-limiting toxicity
ECOG: Eastern Cooperative Oncology Group
mTOR: Mammalian target of rapamycin
RECIST: Response evaluation criteria in solid tumor
